# Clinical outcomes of new toric trifocal diffractive intraocular lens in patients with cataract and stable keratoconus: Six months follow-up

**DOI:** 10.1097/MD.0000000000006340

**Published:** 2017-03-24

**Authors:** Doroodgar Farideh, Sanginabadi Azad, Niazi Feizollah, Niazi Sana, Alinia Cyrus, Ghoreishi Mohammad, Baradaran-rafii Alireza

**Affiliations:** a Eye Research Center, Tehran University of Medical Sciences; b Eye Research Center, Shahid Beheshti University of Medical Sciences and Health Services, Tehran; c Eye Research Center, Isfahan University of Medical Sciences and Health Services, Isfahan, Iran.

**Keywords:** cataract, keratoconus, toric multifocal intraocular lens

## Abstract

**Purpose:**

To evaluate the clinical results of toric trifocal diffractive intraocular lens in eyes with cataract and mild keratoconus.

**Methods:**

Five keratoconus patients (10 eyes) that had bilateral AT LISA 939 implantation were selected and had followed in 3-time horizons of 1, 3, and 6 months. Patients were 46 to 65 years old age, corneal astigmatism of (2.00 D at 6.75 D) and cataract that all of them needed cataract surgery. The distance, intermediate and near visual acuities, defocus curve, ocular aberrations, contrast sensitivity, were measured as effectiveness criteria.

**Results:**

Average of binocular uncorrected distance visual acuity (UDVA) improved from 0.72 log MAR ± 0.11 (SD) to 0.04 ± 0.04 (*P* < 0.05) log MAR, average of uncorrected binocular intermediate visual acuity (UIVA) (80 cm) improved from 0.52 ± 0.07 log MAR to 0.14 ± 0.04 (*P* < 0.05) log MAR, and average of binocular uncorrected near visual acuity (UNVA) improved from 0.48 ± 0.09 log MAR to 0.02 ± 0.07 (*P* < 0.05) log MAR at 6 months, respectively. Contrast sensitivity testing showed acceptable results, the binocular defocus curve corroborate were in appropriate good visual acuity even at the intermediate distances, by a gentle slope less than log MAR 0.2 at −1.5 D, with regard to the best distance visual acuity at the 0 D defocus.

**Conclusions:**

Trifocal AT LISA 939MP IOLs provided appropriate distances, near and intermediate of the visual results. Prediction of the refractive results and optical performances were good.

## 1 Introduction

Keratoconus is an eye disorder which results in progressive thinning of the cornea; in addition to the progressive anterior protrusion subsequent it can rolled not only in intensive myopic astigmatism but also in asymmetrical irregular astigmatism prompting distorted vision. Considering that keratoconus patients mostly demonstrate some range of astigmatism and create cataracts sooner than of the nonkeratoconus patients.^[1]^ Multifocal intraocular lenses (IOLs) were designed to reducing the glasses dependency after cataract surgery and enhancing some aspects which associated to the quality of life. Many clinical studies demonstrate the significant recovery of uncorrected near visual acuity (UNVA) after the implantation of multifocal IOLs in compared with monofocal IOLs, and providing an acceptable visual performance, without reduction in the levels of uncorrected distance visual acuity (UDVA).^[2]^ Usually bifocal IOLs have been unable in full correction of the intermediate distance which is extremely important for reading desktop and computer work.^[3,4]^ Recently presentation of trifocal optics on multifocal diffractive IOLs was an achievement in refractive results, permitting patients to read more easily between intermediate (80 cm) and far distances and without gap between near (40 cm) and intermediate distances.^[5–7]^ The multifocal implant seems to provide better visual quality with improved modulation transfer function (MTF).^[8]^ The present study, bilateral implantation of an AT LISA 939MP IOL, a new diffractive plate haptic IOL with a toric trifocal designed for cataract eyes with stable keratoconus, was assessed, howbeit intraocular toric multifocal lenses were not usually considered to treat keratoconus, theoretically the results should be useful for elect patients. The aim of this study is to evaluate the comprehensive visual results in keratoconic patients’ for intermediate and near distance of visual acuity (VA) and the efficacy of astigmatism correction, defocus curve, contrast sensitivity, and ocular aberrations of toric trifocal diffractive IOL.

## 2 Materials and methods

The ethical committee approval of the Tehran University was reached before starting our study. Five keratoconus patients signed an informed consent form after receiving a detailed description of this modality of treatment. Inclusion criteria were age 46 to 65 years, 10 eyes with corneal astigmatism of (2.00 D to 6.75 D) and cataract which needed cataract surgery and IOL implantation. The elimination criteria including prior ocular surgery, ocular disease, retinal or optic nerve disease, amblyopia, diabetes patients, and corneal astigmatism lower than 2.00 D, active intraocular inflammation requiring treatment before 1 year, and endothelial cell count (ECC) less than 1200 cells/mm. Before the surgery, complete ophthalmic examinations were down, including measurement of monocular and binocular UDVA, UIVA (80 cm), and CIVA (80 cm), UNVA (40 cm), CNVA (40 cm) with Early Treatment of Diabetic Retinopathy Study (ETDRS) charts. Goldman applanation tonometry, slit lamp (as devised by Jaeger, Haag-Streit, Switzerland) examination, corneal topography Pentacam HR (Oculus, Wetzlar, Germany), biometry (IOLMaster version 4.3, Carl Zeiss Meditec AG), and fundoscopy. Clinical keratoconus was diagnosed by 1 practiced clinician (AS) based on obvious findings of keratoconus characterize (e.g., corneal topography with asymmetric bow-tie pattern with or without skewed axes).^[9]^ All patients had intolerance to rigid gas-permeable lens and need cataract extraction.

We were evaluated refraction stability and keratometry confirmation (<0.5 D change) for 6 months. All eyes had grade I or II keratoconus according to the Amsler–Krumeich classification, based on astigmatism, corneal transparency, corneal power, and corneal thickness^[10]^ and visually significant cataract was defined by any LOCS II grading ≥ 2. After the surgery convention on 1, 3, and 6 months was identical to the preoperative convention. The postoperative convention also included measuring of visual acuity in near, intermediate, and far distances, contrast sensitivity test was performed under mesopic (3 cd/m^2^) and photopic (85 cd/m^2^) conditions using the CVS1000 contrast sensitivity test (VectorVision, Greenville, SC). Ocular aberration was determined for a 6.0 mm pupil with the ray-tracing Aberrometer (iTrace, Tracey, Technologies, Houston, TX). Participants fixated a near-infrared point light source during measurements. The binocular defocus curve was established and used a defocalization lens from +2 to −3.50 D and performed the best correction for distance by an increment of 0.5 D.

### 2.1 Intraocular lens

Two type of IOL including: nonpreloaded M type from a spherical power of +28.5 to +32.0 D in 0.5 D increase and a cylindroid power of +4.5 to +12.0 D in 0.5 D increase and a preloaded MP type from a spherical power of −10.0 to +28.0 D in 0.5 D increase and a cylindroid power of +1.0 to +4.0 D in 0.5 D increase. The manufacturer's A-constant for this lens is 118.8. The most available method for calculating the trifocal toric IOL is by using the manufacturer's online calculator ZCALC.

### 2.2 Surgery

All of the operations were performed with the same expert surgeon (FD) by using the sutureless temporal incision 1.8 mm and anesthesia drops were used for the patients preceding the surgical procedure. After capsulorhexis and phacoemulsification, the in the capsular bag IOL was implanted through the main correction index by using the Bluemixs 180 injector (MP) (Carl Zeiss Meditec AG). We have prepared 4 limbal reference markers at the 3, 6, 9, and 12 o’clock positions for the patient in supine position and a preoperative marker to avoiding cyclorotations during surgery. IOL position was marked by sterile Mendez gauge regarding to steep corneal meridian. After IOL implantation, the ophthalmic viscosurgical device below the IOL was completely removed by using bimanual irrigation/aspiration cannulas. Finally the alignment of IOL was rechecked by a Mendez gauge. Postoperative topical therapies were a combination of topical antibiotic and steroid.

### 2.3 Statistical analysis

The Kolmogorov–Smirnov test used to check the data distributions normality.

Since the parametric analysis was possible, the Student *t* test and 1-way ANOVA tests respectively for 2 and more than 2 paired groups were performed to comparisons of all parameters between preoperative and postoperative examinations as well as consecutive postoperative visits. Otherwise, since the parametric analysis was not possible, the Wilcoxon rank sum test was applied to evaluate significant differences between examinations. All *P* values quoted were 1-tailed and were presumed statistically significant when the values are below 0.05. All performed statistical analyses were used Stata12 (StataCorpLP, College Station, TX) statistical package.

## 3 Results

The study included 10 keratoconus eyes of 5 patients; average of age was 53.4 ± 6.65 years (range 46–65 years). Average of spherical error was −3.35 ± 1.71 D (range: −1.75 to −6.75 D), and cylindrical error was −3.75 ± 1.37 D (range: −2.00 to −7.00 D) and spherical equivalent of the population −5.2 ± 1.49 D (range: −4.00 to −8.25 D).

### 3.1 Visual acuity and refraction

**Figure 1:**
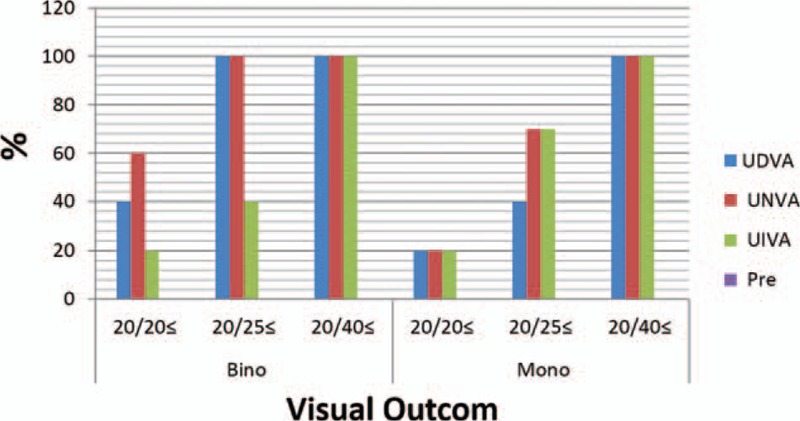
Repartition of binocular and monocular preoperative and postoperative uncorrected distant, intermediate, and near visual outcomes in the analyzed sample.

Without any intra- or postoperative complications such as endophthalmitis, posterior capsule rupture, or corneal decompensation considerable improvement was observed postoperatively in log MAR UDVA, CDVA, UNVA (40 cm), UIVA (80 cm) (*P* < 0.05) (Table 1). Likewise, as expected, a significant decrease in the refractive cylinder was observed postoperatively (*P* < 0.05) (Table 1). All eyes achieved a postoperative refractive cylindrical below 1 D, and 60% of the eyes has showed a postoperative astigmatism of −0.75 D or below. Ninety percent and 50% of eyes showed a 6 months postoperative value within ±1.00 and ±0.50 D, respectively (Fig. 1).

**Table 1:**
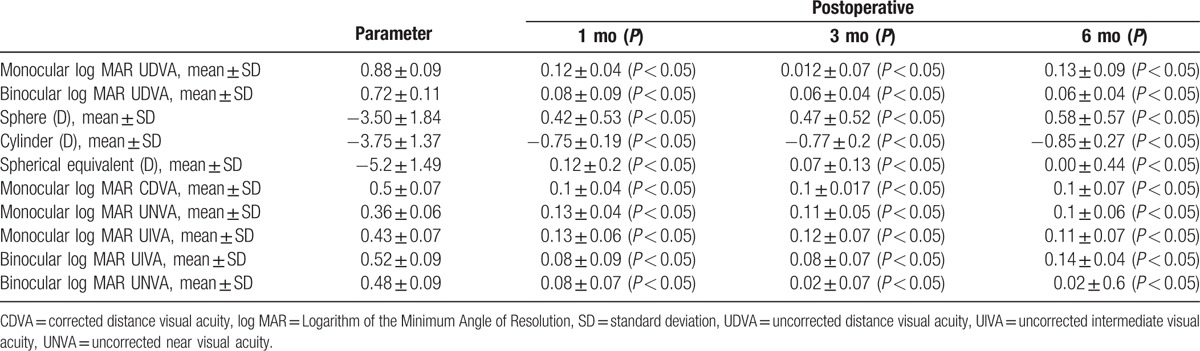
Refractive and visual data in the analyzed during follow up.

### 3.2 Contrast sensitivity

**Figure 2:**
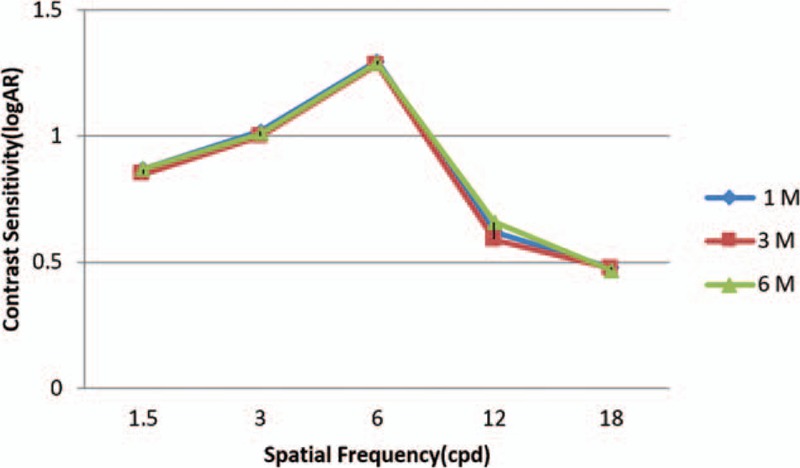
Binocular photopic contrast sensitivity (CS) at 1 (M1), 3 (M3), and 6 months (M6) after surgery.

Figure 2 demonstrates the mean postoperative contrast sensitivity in logarithmic scale under binocular photopic conditions. There was no significant difference in the values obtained at 1, 3, and 6 months. Approximately, values obtained under mesopic conditions were equivalent to those obtained under photopic conditions at all spatial frequency. The curves achieved with monocular vision were equivalent with binocular vision that was achieved.

### 3.3 Defocus curve

**Figure 3:**
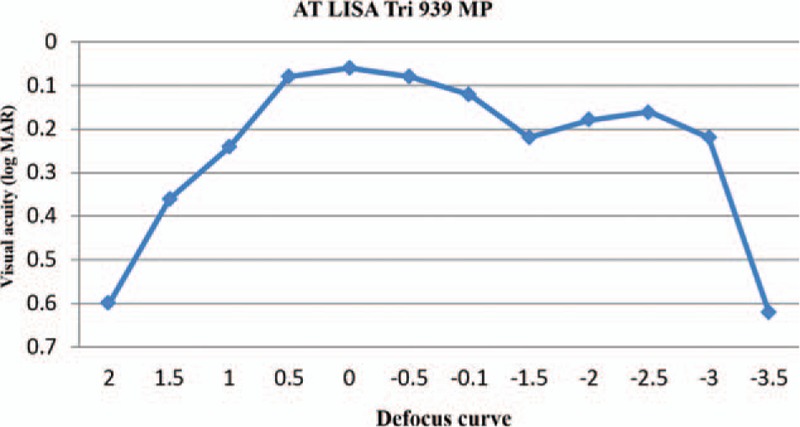
Binocular defocus curve 6 months postoperatively.

Figure 3 demonstrates the mean binocular defocus curve. As shown, functional levels of visual acuity were achieved by the maximum value when any defocus was not presented. Visual acuities better than 0.2 log MAR were observed for defocus levels between +1.00 and −3.00 D (Fig. 3). Defocus curve, log MAR scale, binocular-tested distance corrected.

### 3.4 Aberrometry

**Figure 4:**
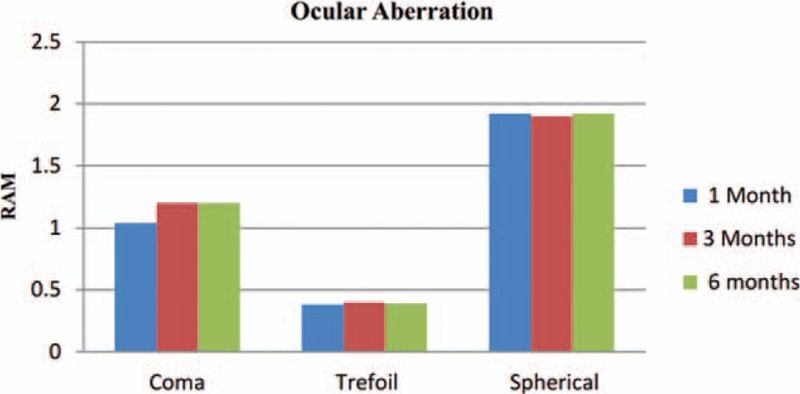
Ocular aberration during 1, 3, and 6 months.

Figure 4 demonstrates mean postoperative the ocular aberration, coma, trefoil, spherical aberrometric data for the present study, respectively. There was not any difference in the values achieved at 1, 3, and 6 months after surgery.

## 4 Discussion

Management of cataract in adult with keratoconus and clear cornea is debated. Recently published approaches have demonstrated that intraocular toric lenses can be utilized to treat permanent keratoconus with promoting outcome.^[11,12]^ To our knowledge, this is the first study that toric multifocal IOL implantation in cataractous eyes with keratoconus is studied with number eyes. We encountered the question whether the multifocal IOLs can give a good vision at different distances for a permanent keratoconus patient who requires cataract surgery and IOL implantation or not.

Designed multifocal IOLs for promoting optical vision in different distances by increasing the depth of field in the eyes.^[13]^ The approach is different according to the characteristic of IOL models.^[14]^ The most common that designed and utilized up to now have been diffractive, refractive, or a combination. Lately, the IOL refractive models being tested and new technologies are being developed. In recent years multifocal IOLs have greatly improved, one weakness of them is disability of providing acceptable range to of vision at the intermediate distance, presentation of trifocal models could promote intermediate vision. Our findings show acceptable visual acuity provided by the IOL in almost all of the distances when tested utilizing the visual acuity test and objectively by blurring vision with defocus addition lenses. In this study, there was recovery in UDVA and CDVA in comparison with preimplantation; thus, trifocal IOL in our study was effective. These outcomes are predictable by obtained the refractive values. All were in the interval of −0.75 to +0.50 (D) of SE 6 months after operation. Montano et al^[15]^ reported a UDVA and CDVA of 20/30 and 20/25, respectively and furthermore patients were satisfied of being glasses independent and have no night vision impairment in the latest case reports. Although they did not report intermediate and near vision while our results obviously impressed a statistical significant increasing of UDVA, UIVA, and UNVA in compared of preoperative results (Table 1). The obtained results of near and intermediate visual in our study were good, as 87% of eyes obtained a log MAR UNVA of ≥0.1, 99% of eyes achieving ≥0.2, and 79% of eyes achieving a log MAR UIVA of ≥0.1, 94% of eyes achieving ≥0.2 and all eyes obtaining ≥0.3 for near and intermediate distance (Fig. 1). Although some patients reported that for reading small handwriting of newspaper, medicine bottles, or food labels need correction. Mojzis et al,^[16]^ Bellucci et al,^[17]^ Kretz et al,^[18]^ Mojzis et al,^[19]^ all reported good average values and demonstrated that toric multifocal IOLs are appropriate selection for the postcataract operation restoration for the far, intermediate, and near visual functions, even though their patients had not keratoconus.

In the present study, the binocular defocus curve demonstrated 2 peaks (at 0.0 D and at −2.5 D) with limited loss of vision within this interval. The worst visual acuity was nearly 0.2 logs MAR, an outcome that further verifies the intermediate visual acuity outcome. It seems that depth of field was 4.9 D (range+1.8 to −3.2 D) for 0.40 log MAR visual acuity, 4.3 D (range +1.2 to −3.1 D) for 0.3 logs MAR, and 4.00 D (range +1.00 to −3.00 D) for 0.25 log MAR. Having a good defocus curve could be due to specific IOL design and attributes and on the residual of astigmatism. This high level of pseudoaccommodation has 2 clinical sequels: first, it may help uncorrected visual acuity in the case of postoperative spherical refractive error. Second, it increases intermediate vision.^[20]^ The depth of field in our study was almost the same to that obtained by Bellucci et al^[17]^ and Visser et al.^[20]^ Three factors have impact on the contrast sensitivity: keratoconus, cataract and, age patients in this study in comparison with the normal population, keratoconus patients have loss of contrast sensitivity.^[21]^ The prior studies demonstrate that contrast sensitivity can increment for old people after operation as an outcome of the elimination of the pacified crystalline lens. However, the aspheric surface theoretically contributes to have better contrast sensitivity, especially under mesopic conditions and optical quality,^[22]^ patients likely need longer following-up to restore contrast sensitivity with diffractive multifocal IOL designs. In our study at 6 months and for 3 cpd, we achieved a mean value of 1.01 ± 0.58 log contrast sensitivity under photopic positions and of 1.01 ± 0.52 log contrast sensitivity under the mesopic positions. The outcomes that we got are like those in different investigations of the toric diffractive multifocal AT Lisa IOL in nonkeratoconic eye.^[13]^ The AT Lisa toric 939 M IOL is the independent pupil; therefore, measurement of contrast sensitivity at the distance focus have no change by disparate luminance levels. Furthermore, the acquired values with binocular vision were similar to the values of monocular vision and there were no distinctions in the 3 measurements taken at 1, 3, and 6 months. This suggests that visual restoration was complete 1 month and results are acceptable according to age, keratoconus, and subjective patient satisfaction after surgery. Keratoconus prepared significantly higher ranges of corneal and ocular aberrations in comparison with normal eyes.^[23,24]^

Aberrometry after multifocal IOL implantation is not completely reliable.^[25]^ Ocular aberrations are extremely pupil dependent.^[26]^ The multifocal implant seems to provide better visual quality with improved MTF.^[8]^ We have seen convenience of patients without image distortion problems due to acceptable neuroadaptation over time.^[27]^ In outcome there was no difference in the values obtained at 3 and 6 months after surgery, it seems that aberration rehabilitation was complete 3 months (Fig. 4). However, further study is necessary with a larger number of patients to confirm these preliminary findings. In conclusion, the implantation of the trifocal AT LISA toric 939MP IOLs can be useful in eyes with cataract associated by stable keratoconus and provided good visual outcomes in distance, intermediate and near during the first 6 months postoperatively. Postoperative outcomes such as contrast sensitivity and aberration results were comparable with preoperative; according daily activity, age, patient satisfaction, and nature of keratoconus disease by this new IOL technology. Further studies are essential to evaluate the stability of visual outcomes provided by this IOL in the more patients with a long-time follow up. It recommended to ophthalmologists that have special regard to selection of appropriate patients and their motivation for having surgery as these factors can seriously impact on the result and patient satisfaction.
